# Wave 2 strains of atypical *Vibrio cholerae* El Tor caused the 2009–2011 cholera outbreak in Papua New Guinea

**DOI:** 10.1099/mgen.0.000256

**Published:** 2019-02-27

**Authors:** Andrew R. Greenhill, Ankur Mutreja, Dieter Bulach, Matthew J. Belousoff, Marinjho H. Jonduo, Deirdre A. Collins, Monalisa P. Kas, Johanna Wapling, Torsten Seemann, Alice Lafana, Gordon Dougan, Mark V. Brown, Paul F. Horwood

**Affiliations:** ^1^​ Papua New Guinea Institute of Medical Research, Goroka, Papua New Guinea; ^2^​ School of Health and Life Sciences, Federation University Australia, Churchill, Australia; ^3^​ Wellcome Trust Sanger Institute, Hinxton, Cambridge, UK; ^4^​ Department of Medicine, University of Cambridge, Cambridge, UK; ^5^​ Melbourne Bioinformatics, The University of Melbourne, Parkville, Australia; ^6^​ Department of Microbiology, Monash University, Clayton, Australia; ^7^​ School of Medicinal and Health Sciences, Edith Cowan University, Perth, Australia; ^8^​ School of Environmental and Life Sciences, University of Newcastle, Callaghan, NSW, Australia; ^9^​ Australian Institute of Tropical Health and Medicine, James Cook University, Cairns, Australia

**Keywords:** cholera, Asia, Pacific, O1, *V. Cholerae*, Oceania

## Abstract

*
Vibrio cholerae* is the causative agent of cholera, a globally important human disease for at least 200 years. In 2009–2011, the first recorded cholera outbreak in Papua New Guinea (PNG) occurred. We conducted genetic and phenotypic characterization of 21 isolates of *V. cholerae ,* with whole-genome sequencing conducted on 2 representative isolates. The PNG outbreak was caused by an atypical El Tor strain harbouring a tandem repeat of the CTX prophage on chromosome II. Whole-genome sequence data, prophage structural analysis and the absence of the SXT integrative conjugative element was indicative that the PNG isolates were most closely related to strains previously isolated in South-East and East Asia with affiliations to global wave 2 strains. This finding suggests that the cholera outbreak in PNG was caused by an exotic (non-endemic) strain of *V. cholerae* that originated in South-East Asia.

## Data Summary

PRJNA448948: WGS of *Vibrio cholerae* isolates from the 2009–2011 cholera outbreak in Papua New Guinea. Readsets for strains L1 and M2 have been submitted to the SRA under Bioproject number PRJNA448948 (url - www.ncbi.nlm.nih.gov/bioproject/PRJNA448948).

**Impact Statement**

Cholera, a severe watery diarrhoea, has been recognized as an important human disease for hundreds of years. The current (seventh) pandemic continues to cause considerable morbidity and mortality, predominantly in low-income countries where sanitation and hygiene are lacking. In 2009–2011, the first reported outbreak of cholera occurred in Papua New Guinea (PNG), one of the most populated countries in the Pacific region. A sound understanding of the epidemiology of diseases such as cholera is important for management of disease risk. We conducted genetic analyses including whole-genome sequencing on the causative organism, *Vibrio cholerae *, isolated during the PNG outbreak. Whole-genome sequence analysis revealed that a single strain of *V. cholerae* caused the PNG outbreak; and the strain was closely related to strains that were circulating in South-East and East Asia approximately 10 years prior to the outbreak in PNG. This study did not seek to establish a mechanism of incursion of cholera into PNG, and there is no direct evidence of importation of the causative strain directly from any single country in South-East or East Asia to PNG.

## Introduction

Papua New Guinea (PNG) is the largest Pacific Island nation, with a population of ~8 million people. Health and social indicators are poor relative to regional and global averages [1]. Less than half the population has access to improved sanitation and safe water, with no appreciable improvement in recent years [1, 2].

Cholera, caused by *Vibrio cholerae *, remains a major health threat in low-income countries. Despite poor sanitation and hygiene, there are no reports of cholera in PNG prior to 2009 [3]. By the end of the 2009–2011 outbreak, >15 500 cases and ~500 deaths were reported [4, 5]. Similarly to the global situation [6, 7], the number of reported cases may greatly under-represent the true burden of cholera in PNG, attributable to the remoteness [8] and limited diagnostic capacity outside the nation’s capital [9].

The O1 serotype of *V. cholerae *, the major cause of epidemic cholera, is divided into classical and El Tor biotypes based on genetic and phenotypic differences. The seventh (current) pandemic is caused by the El Tor biotype, which displaced the classical biotype responsible for the sixth pandemic. Now only genetic remnants of the classical strain remain through outbreak isolates with a mixture of classical and El Tor genotypic characteristics of the cholera toxin (CTX) prophage; and some mixed phenotypic characteristics [10–12]. Such strains have been referred to as ‘atypical’ strains [13]; and more recently have been assigned CTX-1–CTX-3 [14].

Variable number tandem repeat (VNTR) and multi-locus sequence typing (MLST) analyses suggested that a single clone of atypical El Tor *V. cholerae* caused the PNG outbreak [4]. Here, we used whole-genome sequencing (WGS), molecular characterization of the CTX prophage and plasmid detection by PCR to comprehensively characterize PNG isolates.

## Methods

### Bacterial isolates and phenotypic testing

We analysed 21 isolates of *V. cholerae* collected in five provinces of PNG between January 2010 and April 2011. Twenty isolates were from patients presenting to hospital outpatient departments with acute watery diarrhoea, and one isolate (Env1) originated from a suburban watercourse collected at a time of high cholera transmission in Madang (Table S1, available in the online version of this article). Maps of PNG including provincial names have been provided in previous publications [4, 5]. Phenotypic testing was conducted to gain an insight into the characteristics of PNG isolates (whether they were phenotypically aligned with Classical, El Tor or atypical strains); and to determine antimicrobial susceptibility (Supplementary Text).

### Genetic analyses and genome sequencing

Genomic DNA was extracted from overnight Luria–Bertani broth cultures using a DNeasy blood and tissue kit (Qiagen). Twelve isolates were previously characterized by MLST and VNTR [4]. Nine additional isolates underwent genetic typing using the same methods. The genetic structure of the CTX prophage and other mobile genetic elements was determined using PCR and sequencing, using novel (Table S2) and previously described primers [15, 16]. The sequence type of the *tcpA* gene was determined by PCR amplification using specific primers [15], followed by direct sequence analysis (Macrogen). To confirm the location of the CTX prophage, long-range PCRs were conducted targeting the relevant region of each chromosome (Table S2 and Supplementary Text). Isolates were tested for the presence of the SXT integrative and conjugative element (ICE) using primers targeting the boundary of the insertion point for the SXT ICE (Table S2 and Supplementary Text).

Two representative isolates, L1 from Lae and M2 from Madang (both isolated in 2010), were selected for whole-genome shotgun sequencing based on initial biotyping. Genomic DNA was fragmented and sequenced on an Illumina HiSeq 2000 sequencer (Ramaciotti Centre for Genomics, Sydney, Australia). Details of the genome sequencing are provided in Table S3 and the Supplementary Text.

The paired-end reads for the two PNG isolates and publicly available short-read data from another 222 isolates (Table S4) spanning all the waves of seventh pandemic cholera (wave 1, 2 and 3) were mapped to the reference N16961 El Tor strain (National Center for Biotechnology Information accession numbers AE003852 and AE003853) using smalt (www.sanger.ac.uk/ science/tools/smalt-0). A whole-genome alignment of 222 isolates was obtained and single nucleotide polymorphisms (SNPs) were called using the approach described by Mutreja *et al*. [14]. The reads that did not map to the reference N16961 genome were filtered out during the SNP calling process, and any SNP with a quality score less than 30 was excluded. An SNP was considered true only if at least 75 % of the mapped reads covering the heterogeneous site showed consistent difference to the reference. Maximum-likelihood phylogenetic trees based on the SNPs called and filtered were constructed using the default settings of RAxML v0.7.4. In brief, the general time-reversible model with gamma correction was used for among-site rate variation for ten initial trees and a 1000 bootstrap function was used [17]. Recombinogenic SNPs were excluded using Gubbins [18]. Data from a pre-seventh pandemic strain, M66 (accession numbers CP001233 and CP001234), was used to root the final phylogeny [19], and FigTree (http://tree.bio.ed.ac.uk/software/figtree/) was used to visualize and order the nodes of the final robust phylogenetic tree. To cross-check antimicrobial-susceptibility patterns obtained using phenotypic methods (Table S1) with WGS data, we checked against Resfinder [20] using Abricate (https://github.com/tseemann/abricate).

## Results

All isolates were positive for the classical type *rstR* gene; and negative for the El Tor, environmental and O139 *rstR* genes. Long-range PCR confirmed duplicate CTX prophages (~15 000 bp product) arranged in tandem on chromosome II of all isolates. Analysis using PCR revealed all PNG isolates had an identical CTX prophage profile. Table S5 provides a summary of the phenotypic and genotypic traits and CTX prophage structure of the PNG isolates. Characterization of the *tcpA* gene and the CTX prophage demonstrates that the PNG isolates are ‘atypical El Tor’ at the genotypic level. Thus, the CTX prophage present in the PNG isolates is a hybrid El Tor/classical CTX prophage; classified as CTX-2 by Mutreja *et al*. [14].

**Fig. 1.**
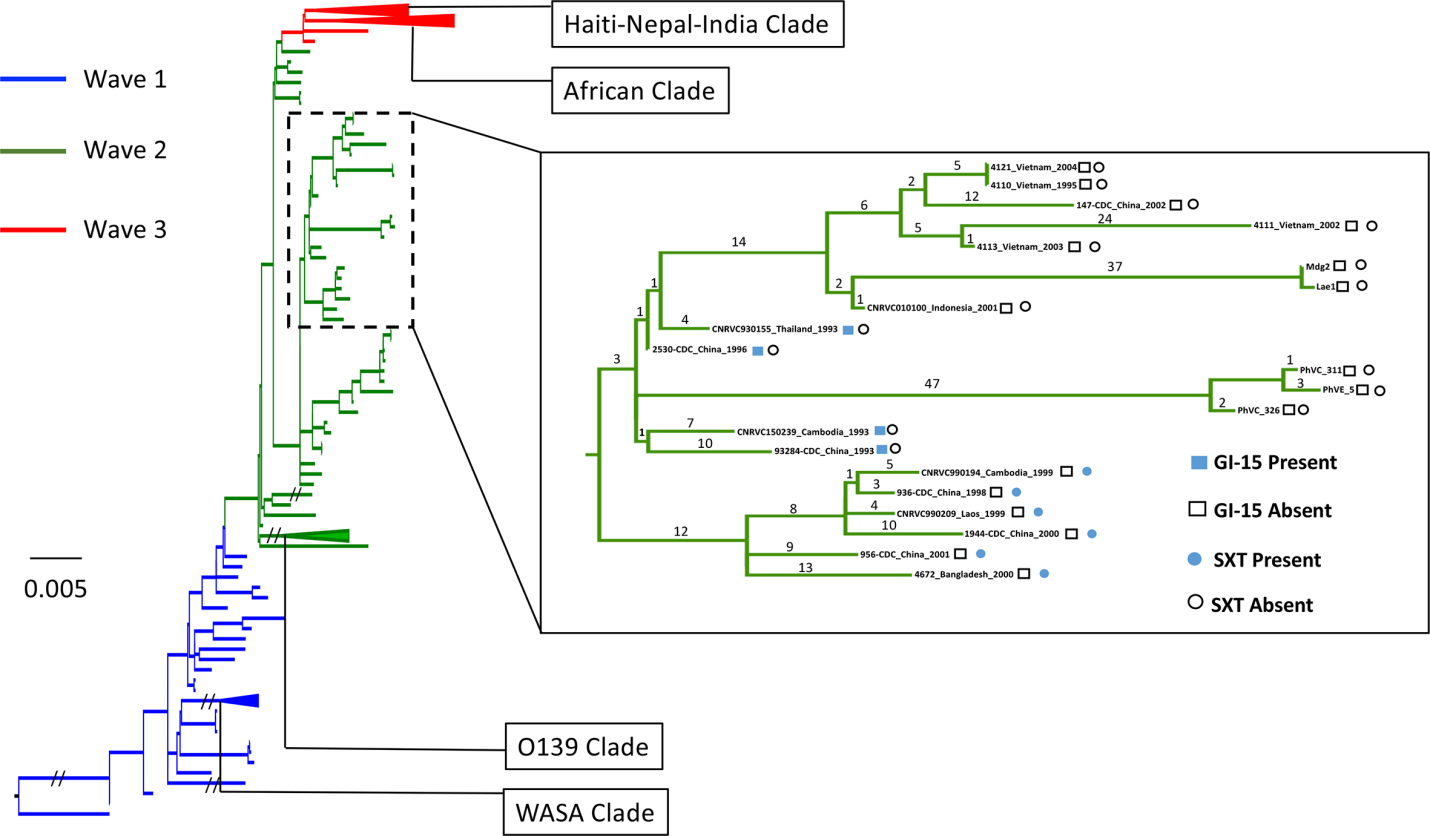
Phylogenetic tree showing the relationship between the recombination-free core genomes of 225 *V. cholerae* strains, including the two PNG isolates (L1 and M2). A total of 7009 SNPs was identified (most attributable to the differences between the pre-seventh pandemic root isolate M66 and the seventh pandemic linage), of which 4966 were assessed as high clustering and therefore removed as recombination. The phylogeny was then based on the remaining 2043 SNPs. The tree was inferred using FastTree. Isolates in the inset containing the SXT insert all had the wave 2 South Asian variant. Bar, number of substitutions per site.

Two representative PNG isolates (L1 and M2) were selected for WGS, enabling more comprehensive core genome comparison with other pandemic *V. cholerae* isolates previously analysed [14, 21, 22]. The PNG isolates were compared to strains from previous studies, based on phenotypic and genotypic (VNTR and CTX profile) characterization and geography (country of isolation) [14, 21–23] (Table S4). In the whole-genome SNP-based phylogeny, the two PNG isolates L1 and M2 clustered with the wave 2 isolates of the seventh pandemic and were closest to an isolate (CNRVC010100) collected from Indonesia in 2001 (Fig. 1). Thirty-eight SNPs separated the Indonesian isolate and the two PNG isolates. The two PNG isolates differed by only a single SNP, at position 872 604 of the reference N16961 genome (in coding region VC_0815). The closely related Vietnamese and Chinese isolates spanned the years 1995–2004. Considering the already established natural molecular evolution rate of 3.3 SNPs per year per genome in the seventh pandemic *V. cholerae* [14], the 37 SNPs suggest that the PNG isolates delineated from their common ancestor with the Indonesian isolate approximately 11 years previously. This corresponds with the time between circulation of wave 2 strains in South-East and East Asia (late 1990s–early 2000s) and the commencement of the PNG outbreak in 2009.

Analysis of the reads that did not map to the N16961 genome sequence revealed the presence of a 34 kb plasmid. This plasmid was absent in the M2 isolate. The presence of the plasmid in isolates that did not have WGS conducted was determined by PCR (Table S2). All isolates other than L1 were negative for the plasmid. Predicted genes present on the plasmid are provided in Table S6.

The PNG isolates L1 and M2 did not harbour the SXT or GI-15 insertions, and antimicrobial-resistance genes were not detected in these isolates. The 14 PNG isolates tested were mostly susceptible to the eight antibiotics examined (Table S1 and Supplementary Text), although antibiotic-resistance profiles of the isolates exhibited some variations (e.g. isolate L6 was resistant to chloramphenicol and tetracycline). The genetic mechanisms underlying these variations are unknown, due to the lack of genome sequence and gene expression data.

## Discussion

Three waves of the current seventh pandemic of cholera have been proposed [14]. Here, we used WGS to demonstrate that the *V. cholerae* O1 strain responsible for the 2009–2011 PNG cholera outbreak is most closely related to strains isolated in Indonesia, Vietnam and China. The related Asian strains circulated between 1995 and 2004, belonging to the second wave of cholera transmission within the seventh pandemic. The relatedness of the PNG strain to strains that previously circulated (1995–2004) in the South-East and East Asia regions is further supported by the shared CTX prophage structure and arrangement. Vietnamese strains 4111 and 4121 have the same arrangement as PNG isolates (no CTX on chromosome I and tandem CTX-2 on chromosome II); while Vietnamese strains 4110 and 4113 also have tandem CTX-2 on chromosome II, with an additional CTX-2 on chromosome I. The lack of the SXT ICE (corroborated in the PNG isolates by a lack of resistance to relevant antibiotics; Table S1) and GI-15 antibiotic-resistance elements is further evidence of relatedness to East and South-East Asian isolates, as wave 2 isolates from other global regions often contain one or both these genetic elements. Notably, isolates closely related to the PNG isolates (Fig. 1) from Indonesia, Vietnam, Laos and China also lack the GI-15 element.

In our comparison with 222 previously sequenced isolates, the PNG isolates L1 and M2 were closely related to an Indonesian strain CNRVC010100 (isolated in 2001) and Vietnamese strains 4110, 4111, 4113 and 4121 (isolated in 1995, 2002, 2003 and 2004, respectively), with a minimum difference of 38 SNPs between the PNG isolates and other isolates from the South-East Asia region. Inadequate surveillance in the Asia–Pacific region, and globally, hampers our understanding of the epidemiology of cholera. Thus, it is not possible to ascertain how the strain arrived in PNG, or where it was persisting prior to the PNG outbreak.

It is now recognized that the incursion of cholera in Haiti was likely the result of human carriers coming to Haiti to contribute to the humanitarian response [24, 25]. Incursions and outbreaks have occurred elsewhere as part of the ongoing seventh pandemic, including South America and Africa [21, 26]. No disaster preceded the PNG outbreak, and there are no obvious epidemiological factors to link the index case or geographical origin of the PNG outbreak to human travel. The Haiti and PNG outbreaks (along with other large outbreaks of the current pandemic [21, 26]) are a stark reminder that the global dissemination of cholera is multifaceted and ongoing, and the disease continues to be a major health and financial burden in settings with poor sanitation and hygiene.

### Conclusion

The genetic relatedness of the PNG isolates to each other (aside from the plasmid) and to regional strains indicates that cholera arrived in PNG through a single incursion, most likely from South-East Asia, that then spread throughout lowland areas.

## Data Bibliography

1. Mutreja A, Kim DW, Thomson NR, Connor TR, Lee JH, *et al*. Evidence for several waves of global transmission in the seventh cholera pandemic. *Nature* 2011;477:462–465. https://doi.org/10.1038/nature10392 (2011). Table S1.
2. Weill FX, Domman D, Njamkepo E, Tarr C, Rauzier J, *et al*. Genomic history of the seventh pandemic of cholera in Africa. *Science* 2017;358:785–789. doi: 10.1126/science.aad5901 (2017). Table S1.
3. Didelot X, Pang B, Zhou Z, McCann A, Ni P, *et al*. The role of China in the global spread of the current cholera pandemic. *PLoS Genet* 2015;11:e1005072. doi:10.1371/journal.pgen.1005072 (2015). Table S1.
4. Klinzing DC, Choi SY, Hasan NA, Matias RR, Tayag E, *et al*. Hybrid *Vibrio cholerae* El Tor lacking SXT identified as the cause of a cholera outbreak in the Philippines. *MBio* 2015;6:e00047-15. doi: 10.1128/mBio.00047-15.

## Supplementary Data

Supplementary File 1
