# Supplementary material for: Wave 2 strains of atypical Vibrio cholerae El Tor caused the 2009–2011 cholera outbreak in Papua New Guinea
Source: Microb Genom. 2019 Feb 27;5(3):e000256. doi: 10.1099/mgen.0.000256 (PMC6487313; doi:10.1099/mgen.0.000256)
Supplement: Supplementary File 1 [file mgen-5-256-s001.pdf]

Supplementary Materials

Detection of Genetic Insertions

CTX prophage location: The primers for chromosome I were designed to amplify the region between locus tags VC1451 and VC1479 (strain N16961, NC_002505 (Chr 1)), while the primers for chromosome II amplified the region between locus tags VCA0569 and VCA0570 (strain N16961, NC_002506 (Chr 2)). Long-range PCR amplification of the CTX region was carried out using the Roche Expand Long Template PCR System^{TM} using primers to determine the presence of CTX prophage on chromosome I (C1ctxF - AGTGCGATTGACGCTCTGGTTCAAGCGATGAGTGG and C1ctxR - TGATTGGCCAGAGAGGTACATAAGAGTTCAAGTGG); and chromosome II (C2ctxF - CTACCCACATTAACTCACGCTGAACAGCAAGTCGC and C2ctxR - GAATCGAAAGGACACAAAGTCAAATTAGCAGGTGG). Standard agarose gel electrophoresis and DNA staining methods were used to determine the size of amplicons and for the estimation of DNA quantity. Sequencing was performed on an Applied Biosystems instrument using a standard Sanger sequencing protocol (Macrogen, Seoul, Korea).

SXT ICE: In the *V. cholerae* wave 2 and 3 strains, the SXT ICE element is inserted into the *prfC* gene (strain N16961, NC_002505 (Chr 1)). Primers designed to flank the insertion point will amplify a product of 1.1 kb if the SXT ICE is absent. Blast was used to confirm the absence of SXT ICE using previously sequenced *V. cholerae* SXT ICE elements as query (e.g. GQ463140- GQ463143). Primers are listed in Table S1.

The SXT ICE was absent in all isolates, as determined by PCR amplification of the region of the genome spanning the insertion point of the SXT ICE (producing a 1.1 kb product for all isolates).

Sequence Analysis

Sequencing libraries were prepared using the Illumina TruSeq library preparation kit and run on the HiSeq 2000 Illumina sequencing instrument.

Raw reads were assembled using *de novo* assembler Velvet v0.7.03 as described in Mutreja et al. 2011 (reference 14 in main text). The Wellcome Trust Sanger Institute hosted genome viewing and comparing tools Artemis and Artemis Comparison Tool (ACT) were used further analyse the genomic content. A summary of read data and the *de novo* assembly is provided in Table S5.

The presence/absence of GI-15 and SXT was confirmed using ACT and manually comparing the assembled genomes against the reference strains. For the detection of GI-15, methods described by Chun et al (1) were used (refer to insertion points for GI-15 provided in Table S1 of Chun et al (1), relative to *V. cholerae* N16961). For detection of SXT, *V. cholerae* O139 strain MO10 (accession number AY055428) was used, as conducted by Mutreja *et al*, 2011 (reference 14 in main text).

The Velvet assembly graph for the Lae read set revealed a circularised contig of approximately 34 kb. The trimmed circularised plasmid sequence was 33,886 bp in length.

Phenotypic Testing

Biotyping: Phenotypic characteristics associated with *V. cholerae*, namely the Voges-Proskauer reaction, susceptibility to polymyxin B (50U) and chicken erythrocyte agglutination, were determined using established procedures (2).

Each of 21isolates of *V. cholerae* was characterized to determine biotype and all had characteristics of O1 El Tor strains, i.e. positive for the Voges-Proskauer reaction, resistant to polymyxin B and positive agglutination of chicken erythrocytes (Table S2).

Antimicrobial susceptibility testing: Susceptibility to selected antibiotics was conducted using the Kirby-Bauer disc diffusion method following published standards for ampicillin, chloramphenicol, tetracycline and trimethoprim-sulfamethoxazole (3, 4). Where interpretive zones were not available for *V. cholerae* (gentamicin, ciprofloxacin, streptomycin and cefotaxime), we referred to the Clinical and Laboratory Standards Institute interpretive zones for Enterobacteriaceae (3). The control strain used was *Escherichia coli* ATCC25922. This is consistent with methods of Sjölund-Karlsson et al (5) in analyzing antimicrobial susceptibility of *V. cholerae*, and is also the origins of interpretive zones provided for ciprofloxacin in the relevant WHO manual (4).

The antibiotic resistance profile of the isolates varied, although the overall trend was that PNG isolates were mostly susceptible to the antibiotics tested (Table S1). Among the fourteen isolates tested, six had reduced susceptibility (intermediate resistance) to ampicillin. One isolate, L6, exhibited multidrug resistance (chloramphenicol and tetracycline). All isolates were susceptible to trimethoprim-sulfamethoxazole, streptomycin and ciprofloxacin.

Table S1: Origins, year of isolation, phenotypic characteristics and antimicrobial susceptibility results of 21 isolates of *V. cholerae* from the PNG cholera outbreak.

Isolate	Year	CCA	VP	Poly B	Amp	Ctx	Cip	Tet	TrS	Gn	St	Chl
Morobe Province												
L1	2010	+	+	R	S	S	S	S	S	S	S	S
L2	2010	+	+	R	I	S	S	S	S	S	S	S
L3[c]	2010	+	+	R	-	-	-	-	-	-	-	-
L4[c]	2010	+	+	R	I	S	S	S	S	S	S	S
L5[c]	2010	+	+	R	-	-	-	-	-	-	-	-
L6[c]	2010	+	+[a]	R	S	S	S	R	S	S	S	R
Madang Province												
M1	2010	+	+	R	S	S	S	S	S	S	S	S
M2	2010	+	+	R	I	S	S	S	S	S	S	S
M3[c]	2010	+	+	R	-	-	-	-	-	-	-	-
M4[c]	2010	+	+	R	-	-	-	-	-	-	-	-
M5[c]	2010	+	+	R	-	-	-	-	-	-	-	-
M6[c]	2010	+	+	R	-	-	-	-	-	-	-	-
Eastern Highlands Province												
G1	2010	+	+	R	S	S	S	S	S	S	S	S
G2	2010	+	+	R	I	S	S	S	S	S	S	S
East Sepik Province												
W1	2010	+	+	R	I	S	S	S	S	S	S	S
W2	2010	+	+	R	I	S	S	S	S	S	S	S
Central Province/National Capital District												
P1	2011	+	+	R	S	S	S	S	S	S	S	S
P3	2011	+	+[a]	R	S	S	S	S	S	S	S	S
P4	2011	+	+[a]	R	S	S	S	S	S	S	S	S
P5	2011	+	+	R	S	S	S	S	S	S	S	S
Env1[b,c]	2010	+	+	R	-	-	-	-	-	-	-	-

[a] Weak positive; [b] Env environmental isolates, from Madang Province; [c] Isolates new to this study. All other isolates (L1, L2, M1, M2, G1, G2, W1, W2, P1, P3, P4, P5) were described and partially characterised in Horwood et al, 2011 [4, main article]

CCA Chicken erythrocyte agglutination; VP Vogues Proskauer; Poly B polymyxin B (50 U) susceptibility; Amp ampicillin; Chl chloramphenicol; Cip ciprofloxacin; Tet tetracycline; TrS

trimethoprim-sulfamethoxazole; Gn Gentamicin; St streptomycin; Ctx cefotaxime; S sensitive; I intermediate resistance; R resistant; - not tested as isolates lost viability.

Table S2: Primer sequences and outcomes for detection of the SXT ICE and characterisation of the CTX prophage for PNG *V. cholerae* isolates. All 21 isolates yielded the same result for a given test, except for plasmid detection.

	Forward primer	Reverse primer	PNG strains
**SXT insertion**	(this study)		
Ch1-sxt	TCCACATCGATTGAAACGTCGACGCGAGCTCACTG	AATTGCATAACAGAGGTGGTCACCGAAATACCACG	negative
**Chromosome**			
**CTX**	(this study)		
Ch1-ctxtlc	AGTGCGATTGACGCTCTGGTTCAAGCGATGAGTGG	TGATTGGCCAGAGAGGTACATAAGAGTTCAAGTGG	negative
Ch2-ctxctx	CTACCCACATTAACTCACGCTGAACAGCAAGTCGC	GAATCGAAAGGACACAAAGTCAAATTAGCAGGTGG	positive
**Plasmid**			
Plasmid parB	GGTGGTTATGTCATTCAGAACTAGG	CGTCGATGATAAGCTCATTACTCTC	negative*
Plasmid virB4	AAACGAATTACAACAGTCTCTTTGC	TCACATCTAAAATTACGCTTTCACC	negative*

* All isolates negative except L1

Table S3: Draft genome sequence overview of PNG isolates L1 and M2.

Read Set						Draft assembly			
Isolate	Reads	Yield (bases)	Average quality	Initial read length	Average trimmed read length	× coverage	Total bases	No. of contigs	Number of protein coding sequences
L1	9,316,148	913,824,831	35.7	101	98	866	3,965,575	99	3490
M2	9,297,168	913,620,593	35.8	101	98	1196	3,931,291	89	3444

Table S4: Phenotypic and genetic characteristics of PNG isolates.

Strains	Country (year)	VNTR profile*	VP	PolyB	CCA	ctxB	RstR	tcpA	rstC	CTX Ch1	CTX Ch2
This study (n=21)	PNG (2009-2011)	10,6,8,(8/9),(11/12)†	Pos	R	Pos	Cl	Cl	ET	Neg	Neg	CTX^{CL}-CTX^{CL}

† The VNTR profiles for 12/21 Papua New Guinea strains are taken from Horwood et al. (4). The additional 9 strains analysed in this study all had a profile of 10, 6, 8, 8 12.

CCA Chicken erythrocyte agglutination; VP Vogues Proskauer; PolyB polymyxin B (50 U) susceptibility; R resistant, S susceptible; Cl Classical; ET El Tor.

Table S5: *V. cholerae* strains used to conduct genome comparisons.

Name	Accession	Country of isolation	Year of isolation
4605	ERR018111	India	2007
4656	ERR018112	India	2006
4675	ERR018113	Bangladesh	2001
4679	ERR018114	Bangladesh	1999
4663	ERR018115	Bangladesh	2001
4661	ERR018116	Bangladesh	2001
4660	ERR018117	Bangladesh	1994
4110	ERR019879	Vietnam	1995
4111	ERR019880	Vietnam	2002
4322	ERR019881	India	2004
4642	ERR019882	India	2006
4670	ERR019883	Bangladesh	1991
4672	ERR019884	Bangladesh	2000
4122	ERR019885	Vietnam	2007
BangladeshA330	ERR018119	India	1993
IndiaMBN17	ERR018123	India	2004
IndiaVC51	ERR018124	India	1992
IndiaV212_1	ERR018125	India	1991
IndiaV109	ERR018126	India	1990
IndiaV5	ERR018127	India	1989
BangladeshA488	ERR018128	Bangladesh	2006
IndiaMBRN14	ERR018130	India	2004

IndiaMJ1485	ERR018120	Bangladesh	1994
BangladeshA383	ERR018121	Bangladesh	2002
IndiaMG160	ERR018122	Bangladesh	1991
KoreaA177	ERR018149	Columbia	1992
KoreaA155	ERR018150	Mozambique	1991
KoreaA154	ERR018151	Mozambique	1991
KoreaA152	ERR018152	Mozambique	1991
KoreaA131	ERR018153	India	1989
KoreaA130	ERR018154	India	1989
KoreaA185	ERR018156	Columbia	1992
KoreaA184	ERR018146	Columbia	1992
KoreaA109	ERR018147	unknown	1990
KoreaA180	ERR018148	Columbia	1992
KoreaA186	ERR018158	Argentina	1992
KoreaA201	ERR018167	Argentina	1992
KoreaA200	ERR018168	Argentina	1992
KoreaA241	ERR018169	Vietnam	1989
KoreaA232	ERR018159	Mexico	1991
KoreaA193	ERR018160	Bolivia	1992
KoreaA231	ERR018161	Mexico	1991
KoreaA245	ERR018171	Vietnam	1989
KoreaA481	ERR018175	Djibouti	2007
KoreaA397	ERR018176	Bangladesh	1987
KoreaA390	ERR018177	Bangladesh	1987
KoreaA346	ERR018179	Bangladesh	1994

KoreaA316	ERR018181	Argentina	1993
KoreaA487	ERR018182	Bangladesh	2007
KoreaA483	ERR018172	Djibouti	2007
KoreaA482	ERR018174	Djibouti	2007
IndiaGP152	ERR018188	India	1979
IndiaPRL5	ERR018189	India	1980
IndiaGP145	ERR018191	India	1979
IndiaGP143	ERR018192	Bahrain	1978
IndiaGP140	ERR018193	Malaysia	1978
IndiaGP106	ERR018194	West Germany	1975
IndiaPRL64	ERR018195	India	1992
IndiaPRL18	ERR018185	India	1984
IndiaGP60	ERR018186	India	1973
IndiaIDHO1_726	ERR018187	India	2009
Kenya6180	ERR019286	Kenya	2007
Kenya6210	ERR019290	Kenya	2007
Kenya6201	ERR019291	Kenya	2007
Kenya6197	ERR019292	Kenya	2007
Kenya6196	ERR019293	Kenya	2005
Kenya6195	ERR019294	Kenya	2005
Kenya6194	ERR019295	Kenya	2007
Kenya6193	ERR019296	Kenya	2005
Kenya6215	ERR019297	Kenya	2005
Kenya6214	ERR019287	Kenya	2007
Kenya6191	ERR019288	Kenya	2005

Kenya6212	ERR019289	Kenya	2007
Kenya7682	ERR028066	Kenya	2009
Kenya7687	ERR028074	Kenya	2009
Kenya7686	ERR028075	Kenya	2009
Kenya7685	ERR028076	Kenya	2009
Kenya7684	ERR028068	Kenya	2009
1346	ERR025356	Mozambique	2005
4551	ERR025357	India	2007
4623	ERR025358	India	2007
4593	ERR025359	India	2007
4538	ERR025360	India	2007
4339	ERR025361	India	2004
4121	ERR025362	Vietnam	2004
4113	ERR025364	Vietnam	2003
4585	ERR025366	India	2007
4552	ERR025368	India	2007
4488	ERR025369	India	2006
4784	ERR025370	Tanzania	2009
4600	ERR025371	India	2007
4646	ERR025372	India	2007
4662	ERR025373	Bangladesh	2001
4519	ERR025374	India	2005
4536	ERR025375	India	2007
1326	ERR025367	Mozambique	2005
1672	ERR025377	Mozambique	2005

4698	ERR025379	India	1980
4713	ERR025380	unknown	1973
4714	ERR025381	Angola	1989
4715	ERR025382	Indonesia	1957
4716	ERR025383	Bangladesh	1979
4717	ERR025384	India	1977
4718	ERR025385	Bangladesh	1971
4719	ERR025386	Bangladesh	1979
4720	ERR025378	Peru	1991
4721	ERR025388	Peru	1991
4722	ERR025389	Peru	1991
4723	ERR025390	Peru	1991
4764	ERR025392	Bangladesh	1994
4772	ERR025393	Bangladesh	2006
4771	ERR025395	Bangladesh	2007
MG16226	ERR025396	Bangladesh	1991
VE1	ERR037738	Kenya	2009
VE2	ERR037739	Kenya	2009
VE3	ERR037741	Kenya	2009
KNE3C	ERR117473	Kenya	2010
KNE134	ERR117480	Kenya	2009
KNE134B	ERR117482	Kenya	2009
KNE11B	ERR117484	Kenya	2010
KNE170	ERR117485	Kenya	2009
KNE3G	ERR117490	Kenya	2010

KNEXX	ERR117544	Kenya	2009
KNEXC	ERR117555	Kenya	2009
KNEXXH	ERR117556	Kenya	2009
KNE168	ERR117569	Kenya	2009
KNC145	ERR117571	Kenya	2010
KNC135	ERR117572	Kenya	2009
KNC151	ERR117573	Kenya	2010
KNC8884	ERR117574	Kenya	2010
KNC56	ERR117577	Kenya	2010
KNC8880	ERR117578	Kenya	2010
KNC133	ERR117579	Kenya	2007
KNC64	ERR117580	Kenya	2010
KNC8889	ERR117581	Kenya	2010
KNC149	ERR117582	Kenya	2009
KNC158	ERR117583	Kenya	2010
KNC11	ERR117586	Kenya	2010
KNC144	ERR117587	Kenya	2010
KNC147	ERR117588	Kenya	2010
KNC161	ERR117590	Kenya	2009
KNC146	ERR117591	Kenya	2010
KNC157	ERR117592	Kenya	2009
KNC1888	ERR117593	Kenya	2010
KNC156	ERR117594	Kenya	2009
KNC8885	ERR117595	Kenya	2010
KNC124	ERR117596	Kenya	2009

KNC155	ERR117597	Kenya	2010
KNC143	ERR117598	Kenya	2010
KNC8669	ERR117599	Kenya	2010
KNC231	ERR117600	Kenya	2009
KNC205	ERR117601	Kenya	2009
KNC1583	ERR117602	Kenya	20109
KNC241	ERR117603	Kenya	2009
KNC206	ERR117604	Kenya	2009
KNC233	ERR117605	Kenya	2009
KNC8679	ERR117606	Kenya	2008
KNC1420	ERR117607	Kenya	2008
KNC207	ERR117609	Kenya	2009
KNC8675	ERR117610	Kenya	2009
KNC8572	ERR117612	Kenya	2009
KNC1509	ERR117613	Kenya	2009
KNC8673	ERR114415	Kenya	2009
KNC8678	ERR114416	Kenya	2009
KNC208	ERR114418	Kenya	2009
KNC1709	ERR114417	Kenya	2009
352STDY5570522	ERR386666	Guinea	2012
2010EL_1786	AELH00000000.1	Haiti	2010
2010EL_1792	AELJ00000000.1	Haiti	2010
2010EL_1798	AELI00000000.1	Haiti	2010
B33	ACHZ00000000	Mozambique	2004
CIRS101	ACVW00000000	Bangladesh	2002

Lae1	SAMN08873981	Papua New Guinea	2010
M66	CP001233/CP001234	Indonesia	1937
Mdg2	SAMN08873982	Papua New Guinea	2010
MJ1236	CP001485/CP001486	Bangladesh	1994
MO10	AAKF03000000	India	1992
RC9	ACHX00000000	Keyna	1985
VC1	SRR308665	Nepal	2010
VC2	SRR308690	Nepal	2010
VC3	SRR308691	Nepal	2010
VC4	SRR308692	Nepal	2010
VC5	SRR308693	Nepal	2010
VC6	SRR308703	Nepal	2010
VC7	SRR308704	Nepal	2010
VC8	SRR308705	Nepal	2010
VC9	SRR308706	Nepal	2010
VC10	SRR308707	Nepal	2010
VC11	SRR308708	Nepal	2010
VC12	SRR308709	Nepal	2010
VC13	SRR308713	Nepal	2010
VC14	SRR308715	Nepal	2010
VC15	SRR308716	Nepal	2010
VC16	SRR308717	Nepal	2010
VC18	SRR308721	Nepal	2010
VC19	SRR308722	Nepal	2010
VC20	SRR308723	Nepal	2010

VC21	SRR308724	Nepal	2010
VC22	SRR308725	Nepal	2010
VC25	SRR308726	Nepal	2010
VC26	SRR308727	Nepal	2010
CNRVC010100	ERR1877943	Indonesia	2001
CNRVC150239	ERR1879538	Cambodia	1993
CNRVC930155	ERR1879570	Thailand	1993
CNRVC990194	ERR1879644	Cambodia	1999
CNRVC990209	ERR1879645	Laos	1999
956-CDC	ERR576975	China	2001
936-CDC	ERR576976	China	1998
93284-CDC	ERR576977	China	1993
147-CDC	ERR579085	China	2002
CNRVC030438	ERR1878096	Iraq	2003
CNRVC980060	ERR976561	Cameroon	1998
CNRVC940184	ERR998662	Chad	1994
CNRVC990019	ERR976585	Mozambique	1999
B_64	ERR044797	Mozambique	2004
Zim_25	ERR044799	Zimbabwe	2005
CNRVC030391	ERR1878094	Somalia	2003
1944-CDC	ERR579924	China	2000
2530-CDC	ERR579921	China	1996
1593-CDC	ERR579930	China	1992
1627	ERR025377	Mozambique	2005
PhVC-311	SAMN02352646	Philippines	2011

PhVC-326	SAMN02352647	Philippines	2011
PhVC-5	SAMN02352648	Philippines	2011

Table S6: Predicted genes on the34 kb plasmid of *V. cholerae* isolate L1

**Left**	**Right**	**Gene**	**Locus tag**	**Product**
252	473		vclae_00068	Hypothetical protein
546	758		vclae_00069	Hypothetical protein
869	1090		vclae_00070	Hypothetical protein
1187	1858		vclae_00071	Site-specific tyrosine recombinase XerD
2560	4161	parB1	vclae_00072	Chromosome-partitioning protein parB
4170	4889		vclae_00073	ParA-like protein
4919	5530		vclae_00074	Hypothetical protein
5502	5849		vclae_00075	Hypothetical protein
5978	6151		vclae_00076	Hypothetical protein
6198	6965		vclae_00077	Hypothetical protein
7772	8746		vclae_00078	Hypothetical protein
9009	9296	sinR	vclae_00079	HTH-type transcriptional regulator sinR
9437	9610		vclae_00080	Hypothetical protein
9594	10244		vclae_00081	VirB8 protein
10241	11335		vclae_00082	Type IV secretory pathway VirB9 components
11335	12531		vclae_00083	Type IV secretion system protein virB10
12524	14275		vclae_00084	Hypothetical protein
14281	15183	ptlH	vclae_00085	Pertussis toxin liberation protein H
15194	17155	traG	vclae_00086	Conjugal transfer protein traG
17420	18226		vclae_00087	Hypothetical protein
18234	19085		vclae_00088	Hypothetical protein

19094	19393		vclae_00089	Hypothetical protein
19407	19727		vclae_00090	Hypothetical protein
19724	22078	virB4	vclae_00091	Type IV secretion system protein virB4
22088	22345		vclae_00092	Hypothetical protein
22393	25416		vclae_00093	Relaxase/Mobilisation nuclease domain protein
25397	25840		vclae_00094	Hypothetical protein
26215	26535		vclae_00095	Hypothetical protein
26606	28870	topB_1	vclae_00096	DNA topoisomerase 3
28867	29112		vclae_00097	Hypothetical protein
29234	29680	ssb_1	vclae_00098	Helix-destabilizing protein
29796	29963		vclae_00099	Hypothetical protein
30165	30380		vclae_00100	Hypothetical protein
30448	30594		vclae_00101	Hypothetical protein
30606	31091		vclae_00102	Hypothetical protein
31105	31395		vclae_00103	Hypothetical protein
31422	31691		vclae_00104	Hypothetical protein
31703	32032		vclae_00105	Hypothetical protein
32046	32255		vclae_00106	Hypothetical protein
32269	32619		vclae_00107	Hypothetical protein
32632	32889		vclae_00108	Hypothetical protein
32892	33140		vclae_00109	Hypothetical protein
33294	33452		vclae_00110	Hypothetical protein
33470	33685		vclae_00111	Hypothetical protein

Supplementary References

1. **Chun J, Grim CJ, Hasan NA, Lee JH, Choi SY, Haley BJ, Taviani E, Jeon YS, Kim DW, Lee JH, Brettin TS, Bruce DC, Challacombe JF, Detter JC, Han CS, Munk AC, Chertkov O, Meincke L, Saunders E, Walters RA, Huq A, Nair GB, Colwell RR.** 2009. Comparative genomics reveals mechanism for short-term and long-term clonal transitions in pandemic *Vibrio cholerae*. Proc Natl Acad Sci U S A **106:**15442-15447.
2. **WHO.** 1987. Manual for Laboratory Investigations of Acute Enteric Infections. World Health Organization, Geneva.
3. **CLSI.** 2012. Performance standards for antimicrobial susceptibility testing; twenty-second informational supplement. CLSI document M100-S22. Clinical and Laboratory Standards Institute, Wayne, PA.
4. **WHO.** 2003. Manual for the laboratory identification and antimicrobial susceptibility testing of bacterial pathogens of public health importance in the developing world. World Health Organization, Geneva.
5. **Sjolund-Karlsson M, Reimer A, Folster JP, Walker M, Dahourou GA, Batra DG, Martin I, Joyce K, Parsons MB, Boncy J, Whichard JM, Gilmour MW.** 2011. Drug-resistance mechanisms in *Vibrio cholerae* O1 outbreak strain, Haiti, 2010. Emerg Infect Dis **17:**2151-2154.
